# Systematic identification and analysis of frequent gene fusion events in metabolic pathways

**DOI:** 10.1186/s12864-016-2782-3

**Published:** 2016-06-24

**Authors:** Christopher S. Henry, Claudia Lerma-Ortiz, Svetlana Y. Gerdes, Jeffrey D. Mullen, Ric Colasanti, Aleksey Zhukov, Océane Frelin, Jennifer J. Thiaville, Rémi Zallot, Thomas D. Niehaus, Ghulam Hasnain, Neal Conrad, Andrew D. Hanson, Valérie de Crécy-Lagard

**Affiliations:** Mathematics and Computer Science Division, Argonne National Laboratory, Argonne, IL 60439 USA; Computation Institute, The University of Chicago, Chicago, IL 60637 USA; Horticultural Sciences Department, University of Florida, Gainesville, FL 32611 USA; Microbiology and Cell Science Department, University of Florida, Gainesville, FL 32611 USA

**Keywords:** Gene fusions, *Escherichia coli*, B vitamin pathways, Metabolic modeling, Essential reactions, Bottlenecks

## Abstract

**Background:**

Gene fusions are the most powerful type of *in silico*-derived functional associations. However, many fusion compilations were made when <100 genomes were available, and algorithms for identifying fusions need updating to handle the current avalanche of sequenced genomes. The availability of a large fusion dataset would help probe functional associations and enable systematic analysis of where and why fusion events occur.

**Results:**

Here we present a systematic analysis of fusions in prokaryotes. We manually generated two training sets: (i) 121 fusions in the model organism *Escherichia coli*; (ii) 131 fusions found in B vitamin metabolism. These sets were used to develop a fusion prediction algorithm that captured the training set fusions with only 7 % false negatives and 50 % false positives, a substantial improvement over existing approaches. This algorithm was then applied to identify 3.8 million potential fusions across 11,473 genomes. The results of the analysis are available in a searchable database at http://modelseed.org/projects/fusions/. A functional analysis identified 3,000 reactions associated with frequent fusion events and revealed areas of metabolism where fusions are particularly prevalent.

**Conclusions:**

Customary definitions of fusions were shown to be ambiguous, and a stricter one was proposed. Exploring the genes participating in fusion events showed that they most commonly encode transporters, regulators, and metabolic enzymes. The major rationales for fusions between metabolic genes appear to be overcoming pathway bottlenecks, avoiding toxicity, controlling competing pathways, and facilitating expression and assembly of protein complexes. Finally, our fusion dataset provides powerful clues to decipher the biological activities of domains of unknown function.

**Electronic supplementary material:**

The online version of this article (doi:10.1186/s12864-016-2782-3) contains supplementary material, which is available to authorized users.

## Background

As soon as a handful of whole genomes had been sequenced in the late nineties, the power of using gene fusions to deduce functional associations between gene families was demonstrated [1, 2]. In what is defined here as a true gene-fusion event, gene products which are separate entities in a given genome are joined together in a single multifunctional polypeptide in another genome. Such fusions, which have been called ‘Rosetta stone’ proteins [1], are often found between genes that are functionally related [3], e.g. genes specifying proteins that catalyze consecutive steps in a metabolic pathway, or genes encoding components of molecular complexes. These fusion events are conceptually different from multi-domain proteins, where the individual domains are never encoded separately while retaining the same functional roles [4–6]. For brevity and convenience we refer throughout this article to protein and domain fusions and use protein names although technically it is not the proteins but the genes that are fused.

Fusion identification methods were first developed to predict protein-protein interactions [1, 2] but because fusion events are relatively infrequent, other *in silico* tools have been more widely used for this purpose (see Table 1 in [7] as well as [8] for recent reviews). The use of fusions has, however, been successful in gene function discovery as part of functional association networks. A recent survey catalogued 30 cases where gene fusion analysis led to a correct functional prediction [9], and several more examples can be given just from our own work [10–14]. The analysis of gene fusion and fission events has also turned out to be an effective way to identify deep-branching evolutionary relationships [15–17]. Finally, correct identification of fusion events is critical for assigning accurate functional annotations because many automated function-calling pipelines call only one of the two functional roles encoded by the fused polypeptide [3, 18, 19]. Hence, because of the multiple uses of fusions, many efforts have been made to accurately identify fusion events across an ever-increasing number of sequenced genomes (Table 1).

**Table 1:** Previous analyses of gene fusions

No. of genomes	Organisms analyzed	No. of detected fused proteins	No. of predicted functional linkages**	Ref	Website	Fusion detection method***	Homology or orthology-based? ***
2	EC, SC	-	6,809 in EC 45,502 in SC	[62]	-	Gene fusion (BLAST) & domain fusion (ProDom)	All homologs (5 % most promiscuous domains removed)
3	EC, PH, SC	-	854 in EC 107 in PH; 918 in SC	[63]	-	Gene fusion (BLAST)	All homologs
4	EC, HI, MJ, SC	64	-	[2]	List of fusions ^a^	Gene fusion (BLAST & S-W)	All homologs
17	Bact, Arch	229	-	[64]	-	Gene fusion (S-W)	Orthologs only (BBH)
24	Bact, Arch (+SC)	2,365 (621 families)	-	[65]	-	Gene fusion (BLAST, component overlap <10 %)	All homologs
30	Bact, Arch (+SC)	4,515	-	[3]	DB (not maintained) ^b^; Fusion stats ^c^	Gene fusion (BLAST)	Orthologs only (one link between each COG)
89	Bact, Arch	∼20,000	-	[66]	FusionDB (not maintained) ^d^	Gene fusion (BLAST)	Orthologs only (BBH)
184	Bact, Arch, Eukar	130,229	2,192,019	[25]	Results for download ^e^	Domain fusion (Pfam)	All homologs (promiscuous domains removed)
20	Bact, Arch, Eukar	49	-	[67, 68]	SAFE software; FED DB (not maintained) ^f^	Gene fusion (BLAST)	All homologs (promiscuous domains removed)
30	Bact, Arch	2,490 by MF 5,339 by FT	-	[69]	MosaicFinder; FusedTriplets software ^g^	Gene fusion (BLAST)	Graph topology of seq. similarity network is used for scoring
1,895*	Bact, Arch	user set-dependent, 2,193 in EC	-	[70]	MicroScope ^h^	n/a	Synteny based fusion detection
2,031*	Bact, Arch, Eukar	user set-dependent	-	[24, 71]	String DB ^i^	n/a	n/a
2,291*	Bact, Arch (+SC)	-	2,209,622	[72]	Prolinks ^j^	Gene fusion (BLAST)	All homologs (promiscuous domains removed)
31,442*	Bact, Arch, Eukar	user set-dependent,397 in EC	-	[34, 73]	JGI IMG ^k^	Gene fusion (USEARCH)	All homologs (as in [2])
user set	Eukar	-	user set-dependent	[24]	CODA software ^l^	Domain fusion (Pfam)	All homologs (scoring immune to promiscuous domains)
2	Eukar (HS, SC)	235 in HS; 189 in SC	-	[74]	Domain Fusion DB ^m^	Domain fusion (Pfam)	All homologs (promiscuous domains removed)
1	Eukar (TT)	80 in TT	-	[17]	DeFuser ^n^	Domain fusion (KOG)	Compares N and C termini of query sequence to KOG DB

The Table is modified and extended from Table 1 in Reid et al. [24]

Abbreviations: *DB* database, *MF* MosaicFinder software, *FT* FusedTriplets software, *n/a* information not available, *S-W* Smith-Waterman, organisms, *Bact* Bacteria, *Arch* Archea, *Eukar* Eukaryota, *EC E. coli, HI H. influenza, HS H. sapiens, MJ M. jannaschii*, *PH P. horikshii, SC S. cerevisiae, TT T. thermophila*

* Statistics as of November 2015

** Predicted potential protein-protein interactions (‘functional links’) based on gene fusion events; the actual fused proteins were NOT reported in some studies

*** Two main bioinformatics approaches to identify fusion events were used: whole protein sequence comparisons (‘gene fusion’) or domain family comparisons (‘domain fusion’)

^a^ http://www.nature.com/nature/journal/v402/n6757/extref/402086a0-s2.html

^b^ http://fusion.bu.edu

^c^ http://www.pnas.org/content/98/14/7940/T1.expansion.html

^d^ http://www.igs.cnrs-mrs.fr/FusionDB/

^e^ http://www.ncbi.nlm.nih.gov/pmc/articles/PMC2248599/#S8

^f^ Contact Sofia KOSSIDA (sofia.kossida@igh.cnrs.fr)

^g^ http://sourceforge.net/projects/mosaicfinder/

^h^ https://www.genoscope.cns.fr/agc/microscope/compgenomics/fusfis.php?

^i^ http://string-db.org/

^j^ http://prl.mbi.ucla.edu/prlbeta/

^k^ https://img.jgi.doe.gov

^l^ ftp://ftp.biochem.ucl.ac.uk/pub/gene3d_data/v12.0.0/coda/

^m^ http://calcium.uhnres.utoronto.ca/pi/no_flash.htm

The automated detection of fusions in thousands of genomes is not trivial, and the difficulty derives from the very mechanisms driving protein evolution. Proteins evolve by gene elongation (fusion of duplicated gene copies) [6] or fusion and/or rearrangement of separate domains [20]. A high proportion of proteins in a given genome accordingly contain more than one domain (e.g. 39 % of the proteins in *Escherichia coli* have multiple domains). These multi-domain proteins can be separated into different categories. The first contains cases where the multi-domain protein has only one functional role such as peptidoglycan glycosyltransferase (EC 2.4.1.129); such proteins should not be considered as bona-fide Rosetta stone proteins, as these proteins fail the functional definition of a fusion. Depending on how these are treated in the fusion search algorithm, this category can artificially inflate the fusion count. The second category is the set of modular proteins where functional domains can be found in different combinations. These include the phosphotransferase transport system (PTS) proteins, the ubiquitous ABC transporter families [21], or the two component regulator system families [22] that are very widespread in bacterial genomes. These are technically fusion proteins with the caveat that their different domains belong to large paralogous families whose members differ mainly in the substrate or ligand they recognize. Such ‘promiscuous domains’ lead to many genes that contain multiple non-overlapping domains. These – although technically fusions – are not the most interesting types of fusions and are not part of the third group corresponding to the Rosetta stone proteins defined above, which are the most informative in terms of functional associations.

Previously, fusions have been identified computationally using two primary strategies. In the earliest strategies (Table 1), BLAST or Smith Waterman based sequence alignment algorithms were applied to align all proteins across all known sequenced genomes, systematically identifying every case where two non-homologous proteins in one genome aligned to non-overlapping regions of a third protein in another genome. This third protein would then be labeled a fusion. This approach was applied extensively prior to 2005, when the number of genomes, and by extension known protein sequences, was still relatively small (<100 genomes) (Table 1). Today, there are >60,000 sequenced genomes (7,000 complete), containing >50 million proteins, making this all-versus-all sequence alignment approach infeasible.

Currently, the most common approach involves using Hidden Markov Models (HMM) of protein domains [23] to robustly align a database of unique protein domains against all known proteins and identifying fusions as proteins that align to multiple non-overlapping domains [24]. The use of HMMs in combination with a database of unique domains serves to massively reduce redundancy in the query sequences for this analysis, making this approach computationally tenable even for tens of thousands of genomes and millions of proteins. The challenge in this approach is that it can lead to many false positives, because of the ‘promiscuous domains’ problem discussed above. To eliminate these false positives, two filters are often applied: (i) elimination of ‘promiscuous domains’ that co-occur in many different proteins with many different domains; (ii) elimination of domains that are not a full-length match to a protein in another genome. While these filtering approaches do reduce false positives, they do not eliminate them entirely [25].

Today, significant progress has been made in defining a set of conserved protein domains that covers much of the current genomic diversity [26] and in compiling a large set of consistently annotated genome sequences [27]. In principle, this set could be used to generate a revised dependable fusion dataset. The accessible identification of fusions in modern genome databases presents a great opportunity for statistical and evolutionary analysis of fusion events on a scale and with a depth that has never been previously possible. Fusion events can be classified, categorized, and analyzed for how commonly they occur. Fusion prediction methods can make better use of machine learning approaches, as datasets are large enough now to enable these approaches. Most importantly, the occurrence of fusions can give insights into the functions of the fused domains.

Several hypotheses have been put forward regarding the selective pressures that drive the formation of fusions. The initial postulates were: (i) that in the case of consecutive steps in metabolic pathways, fusions improve kinetic efficiency by favoring channeling of intermediates between fusion partners, and (ii) that in the case of complexes, fusions ensure identical expression levels of the subunits [1, 2, 28]. The channeling hypothesis was recently challenged as simply fusing genes did not promote channeling whereas protein conglomerates did [29]. The fact that the great majority of fusions (~90 %) occur in only one order (i.e. AB, never BA) also suggests that fusions could optimize complex assembly [30]. Finally, it seems likely that fusions reveal cases of instability/toxicity of pathway intermediates that would fit with the recent proposal by Danchin and colleagues that chemical reactivity shapes many aspects of metabolism and cellular structure [31].

In this study we combined the use of the Conserved Domain Database (CDD) [26] and the SEED [32] together with current computational strategies to create an accurate fusion detection algorithm and then a revised dependable fusion dataset. Compared to existing methods summarized in Table 1, our pipeline combined multiple filter criteria and used manually created training sets to fine-tune the parameters to better circumvent the problem of false positives. We focused on prokaryotic genomes because metabolic annotations and models are better for prokaryotes and paralog expansions complicate fusion data for eukaryotes [25]. We also analyzed our updated fusion dataset in order to improve our understanding of where and why gene fusion events have occurred, and of what gene fusions can tell us about the functions of their constituent domains.

## Results

### Compilation of a high quality *Escherichia coli* K12 MG1655 fusion dataset

*E. coli* MG1655 provides an ideal training set for the development of algorithms to identify multi-domain fusions based on protein sequence (Table 1). There are four comprehensive fusion analyses in this organism: (i) Enright et al. [2] identified 24 fusions based on comparison of four genome sequences; (ii) Serres et al. identified 107 fusions based on manual curation of protein domain data [33]; (iii) IMG predicted 461fusions, with 74 listed as curated [34], and (iv) SEED annotated 96 fused proteins [27]. We made a reconciled list of fused genes by comparing and curating these data sources using our own fusion criteria: the multi-domain standard and the independently occurring domain standards.

First, we removed fusions that failed to meet the basic criterion of containing multiple non-overlapping protein domains by computing conserved domains for all the predicted fusions using the Conserved Domain Database (CDD) detection scripts obtained from NCBI [26]. Two genes in the Enright et al. dataset were found to be erroneously classified as fusions due to miscalled genes in *Haemophilus influenzae*. Twenty seven genes in the SEED dataset were actually multifunctional single-domain proteins that were inaccurately annotated as fusions. After removing these mispredictions, 151 distinct genes remained from all fusion prediction datasets in *E. coli* that satisfied the criteria as multi-domain proteins (Additional file 1: Table S1).

As a second test, we determined whether the non-overlapping domain alignments in all the predicted fusions: (i) were full-length alignments to each domain; (ii) had greater than 50 % identity to each domain; and (iii) involved non-overlapping domains that also aligned individually to separate single-domain proteins. These criteria are meant to assess whether these proteins are true Rosetta stone proteins. Manual curation of the 38 genes that failed this test revealed that eight were still likely to be fusions based on literature evidence or domain alignments that only narrowly missed the cutoffs listed above. The other 30 genes were labeled as uncertain fusions.

**Fig. 1:**
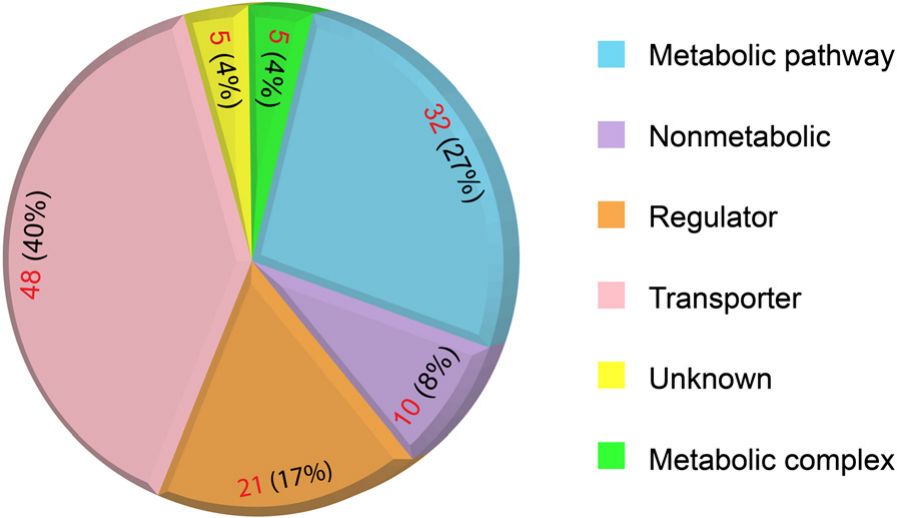
Distribution of functions associated with gene fusion events in *E. coli*. Each of the 121 fused genes identified in *E. coli* was manually assigned to one of six categories based on their annotated function. The distribution of fusions among these categories is shown in the pie chart. Red numbers represent the total fusion counts and black numbers their respective percentages

The 121 fusions that remained after applying these criteria were used as the training set for our fusion prediction algorithm (Additional file 1: Table S1). Thirty genes from this final set were present only in the Serres et al. dataset; 16 genes were present only in the SEED dataset; and none were present only in the IMG dataset. The three dominant functions associated with the fused genes in our *E. coli* dataset were: (i) solute transport, 48 genes; (ii) enzymes in intermediary metabolism, 32 genes; and (iii) regulation, 21 genes (Fig. 1). Ten of the fusions involved non-metabolic functions, and only five were of unknown function. This is a surprising result, as these proportions of transport- and regulation-related fusions do not reflect the functional distribution of *E. coli* genes. Less than 10 % of *E. coli* genes are associated with transmembrane transport [35], yet they represent 40 % of the fusions. Less than 5 % of *E. coli* proteins are regulators [36], but they represent 17 % of the fusions. The number of fusions with enzymes (25 %) is, however, consistent with their genomic representation, which is estimated at 30 % [37, 38]. The small number of fusions involving domains of unknown function in *E. coli* is a tribute to its status as a model organism for over 60 years.

### Manual compilation of fused genes in B vitamin pathways

**Fig. 2:**
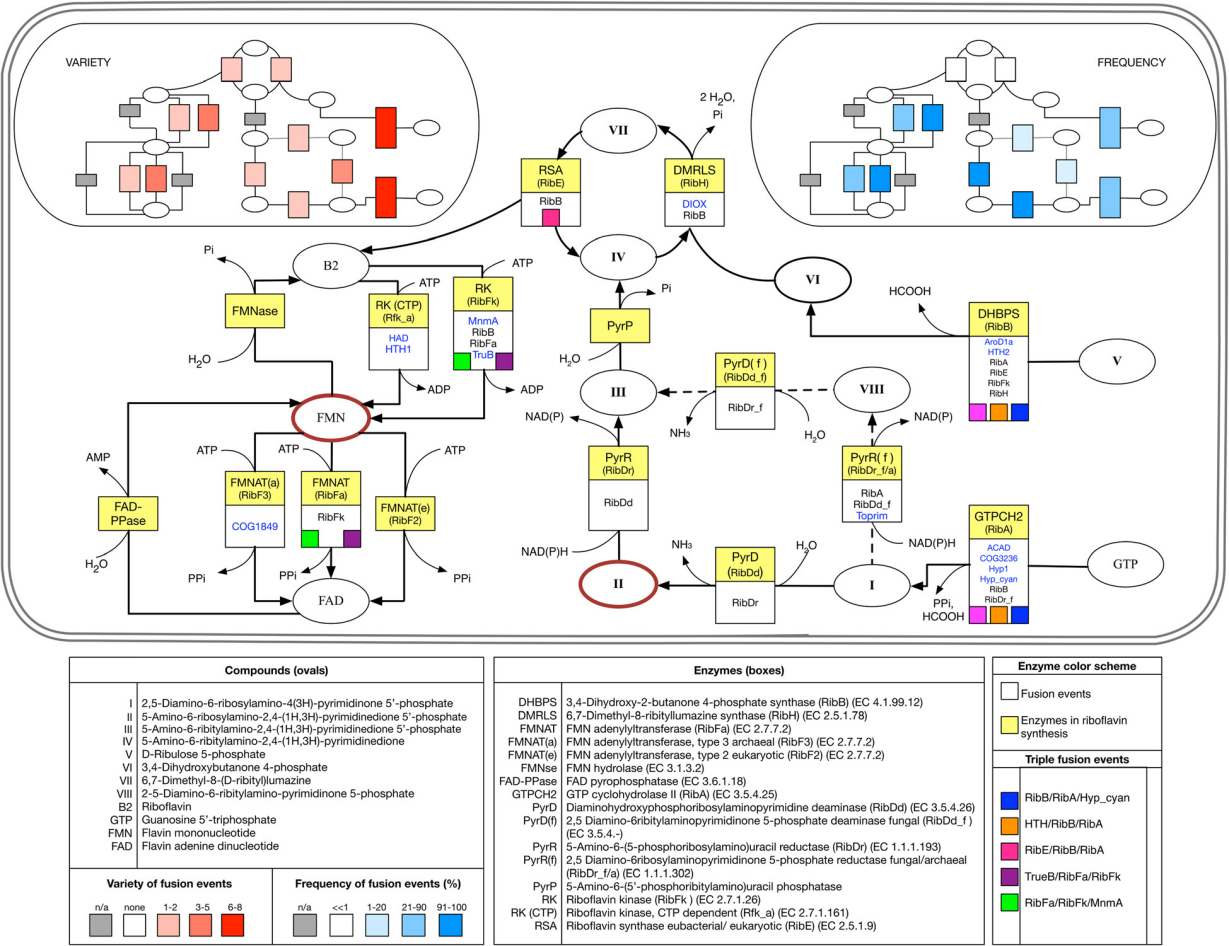
Distribution, variety, and frequency of fusion events in riboflavin biosynthesis. Riboflavin gene fusions and identification are given in (Additional file 1: Tables S2 A and B). Pathway enzymes are shown as yellow boxes and their abbreviations are given in the central bottom panel. The domain name is given in parentheses inside each yellow box. Fusion partners are listed in the white boxes immediately below the corresponding pathway enzyme. Of these, domains of the riboflavin pathway are in black font and unknown domains or domains belonging to other pathways are in blue font. Compounds are shown in ovals. Their abbreviations are given in the left bottom panel. Highly reactive compounds are marked with a red oval. Participation in triple fusion events is flagged with colored squares inserted in the white boxes of the corresponding enzymes. The identification code for these squares is given in the bottom right panel. The variety of fusions of each riboflavin gene is shown on the top left insert. This variety is represented by a color range where the number of binary fusion events in which each gene participates (see Additional file 1: Table S2B), is proportional to the orange color intensity. The frequency of fusions of each riboflavin gene is displayed on the top right insert. This frequency is expressed as a percentage and it was calculated as described in Methods. It is represented by a color range where the ratio for each riboflavin gene mentioned above is proportional to the blue color intensity. Enzymes that participate in only a few fusions are colored grey in both inserts

**Fig. 3:**
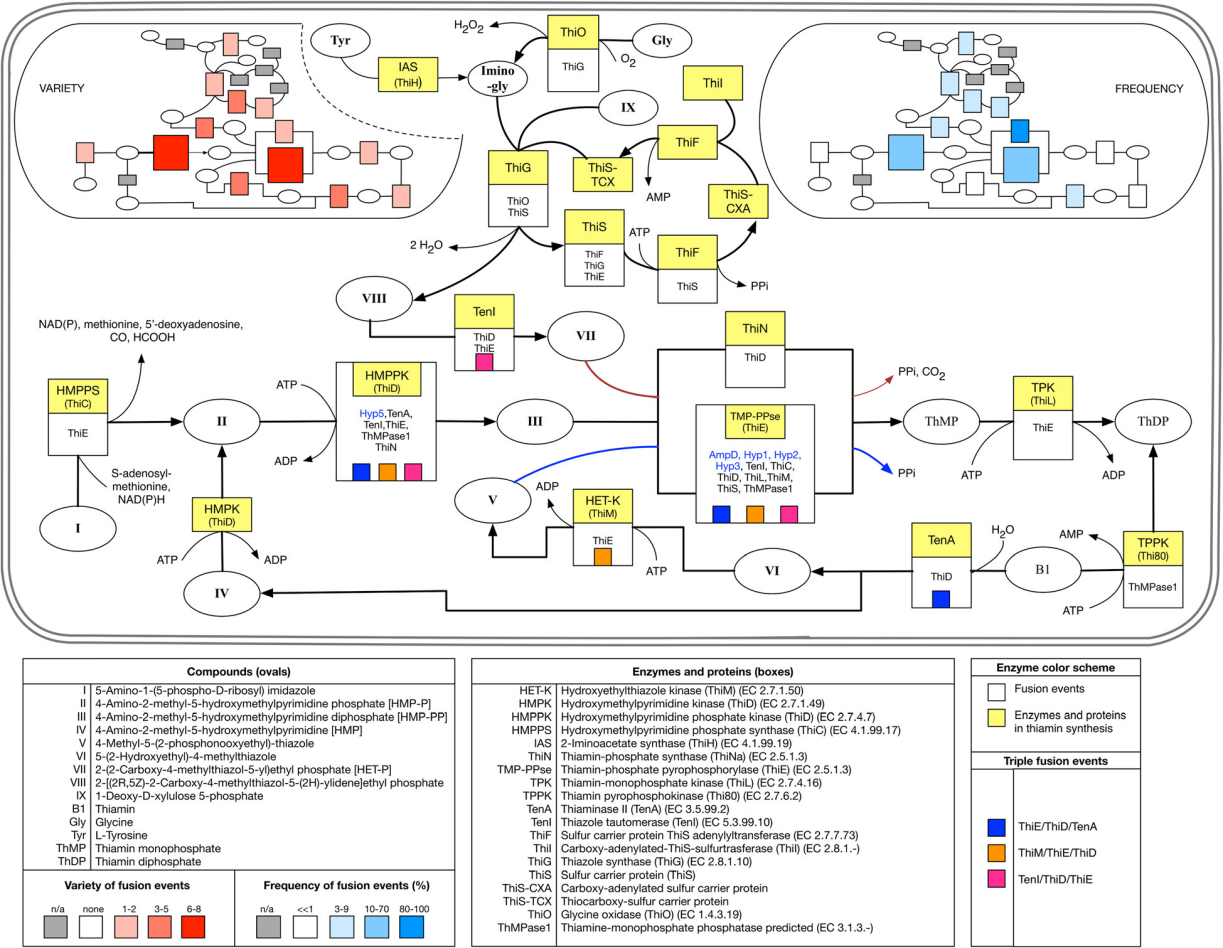
Distribution, variety, and frequency of fusion events in thiamin biosynthesis. The fusion architectures involving these genes as well as their identification data are given in (Additional file 1: Tables S3 A and B). The thiamin biosynthesis pathway enzymes, reactions, intermediates and fusion architectures are illustrated in this diagram following the same representation rules as in Fig. 2. The variety of fusions of each thiamin biosynthesis gene is shown on the top left insert. This variety is represented by a color range where the number of binary fusion events in which each gene participates (see Additional file 1: Table S3B), is proportional to the orange color intensity on the diagram (see left panel under compounds abbreviations section). The frequency of fusions of each thiamin biosynthesis gene is displayed on the top right insert. This frequency is expressed as a percentage and calculated as described in Methods. It is represented by a color range where the ratio for each thiamin gene mentioned above is proportional to the blue color intensity (see right panel under compounds abbreviations section)

Having constructed a comprehensive catalogue of all fusions that occur within a single genome (i.e. *E. coli*), we also wanted to construct a catalogue of all fusions that occur within a single biological system across many different genomes. We selected B vitamin biosynthesis for this detailed cross-genome study because previous work found a high incidence of fusions in these pathways [39]. Since B vitamin enzymes have been well characterized in several prokaryotes, we manually curated three different genome databases (see Methods) and constructed a B vitamin prokaryotic fusions dataset comprising 131 fusions (Figs. 2 and 3). We then used this dataset in combination with our high quality *E. coli* dataset to implement our fusions search algorithm. Vitamin fusion data are compiled in Additional file 1: Tables S2-S8 and Additional file 2).

### Developing an algorithm to systematically detect fusions in all pathways

Most of the recent fusion detection algorithms based on conserved domains (Table 1) have not been applied systematically to a full modern database, and – as shown by our curated analysis in *E. coli* – all of them give high rates of false positives and false negatives. We developed a new fusion detection algorithm, using data from 11,473 genomes and ~42.2 million genes selected from the PubSEED database [27, 32].

We began by using CDD detection scripts obtained from NCBI [26] to identify all instances of CDDs in our genomes. In total, 39,381 unique CDDs aligned to 34.4 million genes (7.8 million genes aligned to no CDDs at all), with an average of 18.9 hits for every gene in our database (Additional file 1: Table S9). In this analysis, any alignment with a BLAST E-value below 1e-5 was considered a hit.

Next, we identified all genes in our database with at least two non-overlapping CDD alignments. This resulted in an average of 1,041 predicted fusions per genome, including 1,654 predicted fusions in *E. coli* (Additional file 1: Table S9). Recall that our manual curation of gene fusion events in *E. coli* identified only 121 fusions in the genome. All of these were among the 1,654 genes with non-overlapping CDD alignments, establishing this condition as necessary but not sufficient for a gene to be considered a fusion. Analysis of selected non-fusions containing non-overlapping CDD alignments revealed that many of these false positives involved CDDs associated exclusively with small sub-domains rather than entire genes.

To eliminate the over-predictions mentioned above, we limited the domains used in our fusion identification approach to CDDs with a bidirectional alignment greater than 90 % to at least one gene in the PubSEED database. This reduced the number of CDDs used for our fusion identification from 39,381 to 26,882 (68 %) (Additional file 1: Table S10). We call these remaining CDDs full-gene-CDDs.

We then narrowed the conditions on our fusion identification algorithm to select only genes with non-overlapping alignments to at least two full-gene-CDDs. We also required the length of the non-overlapping alignments to exceed at least 50 % of the length of the aligned full-gene-CDDs. This 50 % threshold was selected to maximize the fit of our predicted fusions to our curated *E. coli* and B vitamin fusion training set (Additional file 1: Tables S1 to S8). Using these criteria reduced the average fusion count per genome to 686, and the count in *E. coli* to 610 (Additional file 1: Table S9). At the same time, all but ten of our 121 known fusions in *E. coli* were still captured by the more stringent selection criteria. Thus we had eliminated 1,044 false positives in *E. coli* while introducing only ten false negatives.

Another common criterion utilized in fusion prediction algorithms is to exclude “promiscuous” CDDs, i.e. those that are fused to many other domains, when evaluating whether a protein has two non-overlapping domains. Unfortunately, given the size and diversity of our protein database, the majority of CDDs co-occur in many genes with many other CDDs, making all CDDs appear to be promiscuous. We attempted to consolidate CDDs with similar alignments in many different genes into 5,923 distinct sets (Additional file 1: Table S11), where each set contained an average of 6.6 CDDs. However, even with this consolidation, most CDD sets co-occurred in many genes with many other sets, so our efforts to use CDD promiscuity as an additional filter for our fusion identification algorithm failed.

**Fig. 4:**
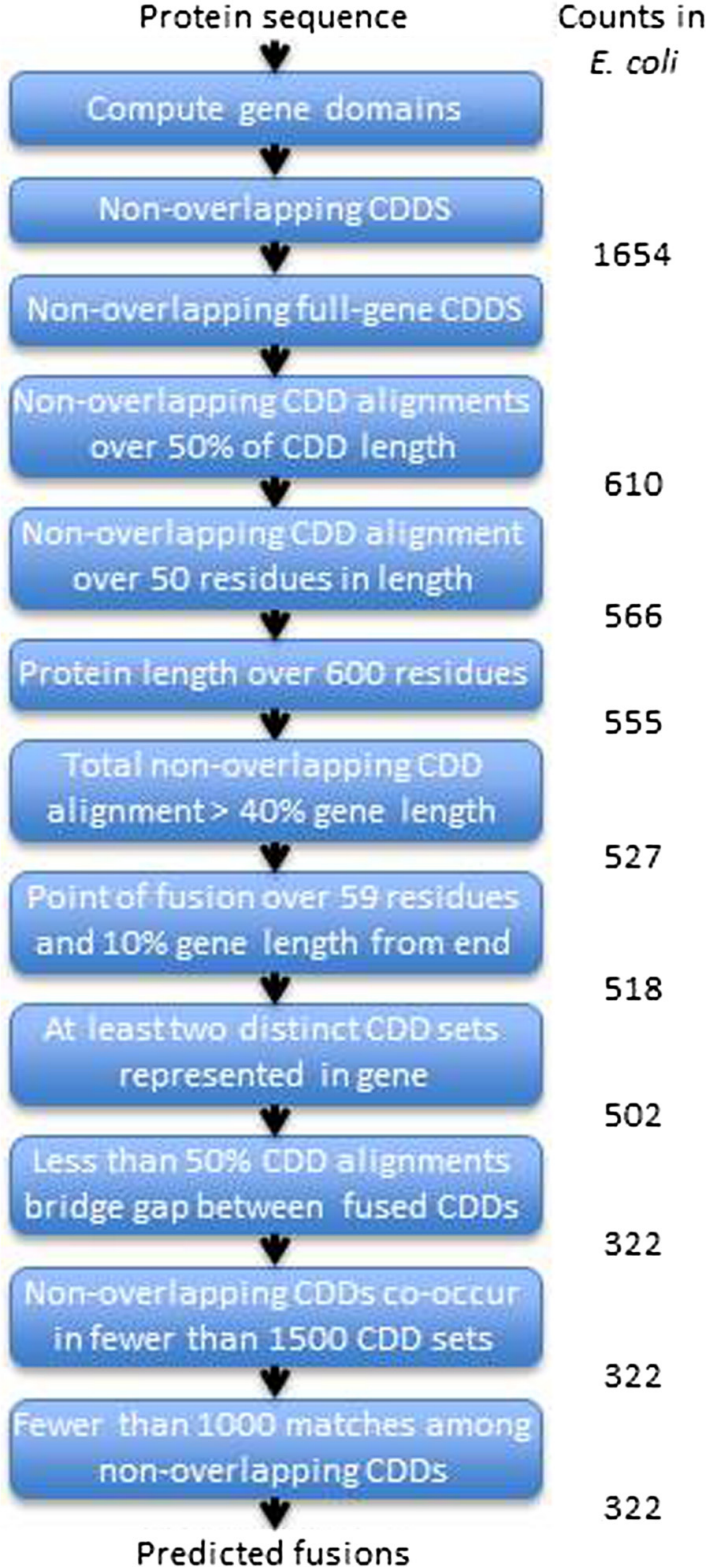
Workflow of our fusion prediction algorithm. Previous protein-domain-based algorithms (see Table 4) overlap with the first three steps of our own algorithm, and other algorithms often include length-based (step 6) or domain promiscuity-based (step 11) criteria. Our algorithm is unique in its application of all these criteria with these specific parameters

Instead of the CDD promiscuity filter, we identified a set of eight alternative criteria that could be used to filter non-fusions from true fusions (Table 2). These criteria were determined by comparing the 121 true fusions to the 499 false positives in *E. coli* that satisfied our non-overlapping full-gene-CDD filter. We identified the biologically meaningful attributes of these genes and their CDD alignments that worked best to separate fusions from non-fusions (Table 2). With these refined criteria, we reduced the predicted number of fusions in *E. coli* to 322, including 98 (81 %) of our 121 confirmed fusions in *E. coli*. Our algorithm also correctly predicted 126 (96 %) of the 131 B vitamin fusions. The workflow of our fusion prediction algorithm is presented Fig. 4.

**Table 2:** Criteria used to filter true fusions from false positives

ID	Criteria	Biological meaning
1	Protein length must exceed 600 amino acid residues	Fusion proteins should be longer than single-domain proteins
2	All non-overlapping CDDs together must align to at least 40 % of the gene length	Fused-domains should cover the full length of the fused gene
3	A minimum alignment length of 50 for all non-overlapping CDDs	Fused-domains should represent entire genes and should not be overly short
4	Gap between fused domains must be at least 60 residues and 10 % of gene length from end of gene	Point of fusion should be fairly centrally located in fused gene
5	At least two distinct CDD sets represented in the gene	Fused domains should not belong to the same CDD
6	Less than half of the CDD alignments for the gene should cross the gap between fused domains	A fused gene should be characterized more as a fusion of multiple domains than as a match to a single domain
7	All non-overlapping CDDs must co-occur with fewer than 1500 different CDD sets	Fused domains should not be overly promiscuous
8	Fewer than 1000 matches among the non-overlapping CDDs	Fused domains should be different from one another

Overall, our fusion prediction algorithm has a false negative rate of 11 % and a false positive rate of 50 % (Additional file 1: Table S12, which contains all multi-domain proteins along with how each protein matched or failed to match our fusion criteria). These results represent extensive optimization of numerical thresholds for our fusion prediction criteria, prioritizing the minimization of false negatives over the minimization of false positives. This represents a significant improvement over existing fusions identification approaches used in SEED or IMG. SEED has a lower false positive rate (28 %) but a much higher false negative rate (43 %); and IMG has a higher false negative rate (38 %) and a higher false positive rate (84 %). Here we emphasize that our training set was instrumental in the development of our fusion prediction algorithm, and the use of such a training set is a major factor that distinguishes our approach from previous methods. We tailored our algorithm repeatedly to improve performance against our curated training set. At times, this led to the rejection of criteria used in previous methods that failed to perform well in our analysis (e.g. filtering promiscuous domains). This approach also led to the development of our eight criteria to filter multi-domain proteins that are fusions from multi-domain proteins that are not, which are unique to our algorithm. The false positive fusions that are still predicted by our algorithm are all multi-domain proteins, but based on our curation, they fail the functional definition of a fusion because the non-overlapping domains they contain are not associated independent separable functions.

### Application of the fusion identification algorithm to all genomes

**Fig. 5:**
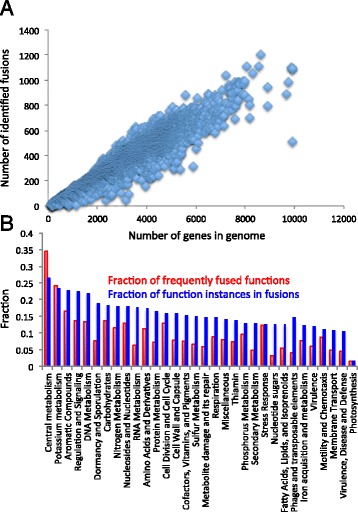
Fusion occurrences across genomes and subsystems. **a** Our fusion algorithm predicted 3.9 million fusions across 11,473 genomes, with the number of fusion events per genome being broadly proportional to the number of genes in the genome. **b** The annotations of these predicted fusions come from a wide range of SEED subsystem classes. Here we show the distribution of predicted fusion events among the 32 prominent subsystem classes. Two distinct measures of fusion prevalence are displayed: (i) the fraction of distinct functional roles in the subsystem that are classified as frequently fused (red bars); and (ii) the fraction of genes associated with any role in the subsystem that are fused (blue bars)

We applied our refined and optimized fusion identification algorithm to our full database of 11,473 genomes and ~42.2 million genes, predicting an average of 338 fusions per genome, and a total of 3,874,379 (11.3 %) fusions overall (Additional file 3 and Additional file 1: Table S9). Comparing the number of genes in each genome versus the number of predicted fusions (Fig. 5a) validates previous observations [25] that the number of fusions is roughly proportional to the number of genes in the genome (~9.1 %).

### Functional analysis of identified fusions using the SEED comparative genomics platform

The SEED platform was created to analyze genomes efficiently and to assign correct annotations to orthologous genes. The strength of the SEED technology is based on the design of its subsystem concept. A subsystem is an ordered collection of functional roles that are related to each other, e.g. as members of a protein complex or as enzymes in a metabolic pathway. A subsystem is linked to a spreadsheet with genomes represented in rows and functional roles in columns. A functional role is defined as the operational task that a gene itself, or its encoded protein, performs in the organism [27, 32].

We conducted a functional analysis of the SEED database, gathering a list of ~253,000 functional annotations assigned to its genes. We focused on the 35,000 functions that were consistently propagated to at least ten genomes within our database and lacked generic descriptors (e.g. predicted, hypothetical, putative, possible, or probable). We found that a mean of 11 % of the genes associated with each functional role were in a predicted fusion. The standard deviation on this mean was quite high at 25 %; this reflected the presence of a small number of functions that were fused far more often than the rest. We specifically identified 2,937 (8.3 %) functional roles where the proportion of fused genes was significantly higher than the mean (t > 2 and *p* < 0.05) at over 61 %. We consider these functions to be *frequently fused* (Additional file 1: Table S13).

Next, we examined the distribution of fusions at a higher level of the SEED annotation ontology, the SEED subsystems. We found that, on average, 14 % of the genes associated with each subsystem were in a predicted fusion (Additional file 1: Table S14 and S15), but some subsystems had a significantly greater percentage of fusions (t > 2 and *p* < 0.05). In 68 subsystems, at least 46 % of the associated genes were classified as fusions. Thirteen of these subsystems were involved in protein metabolism, eight in regulation, six in carbohydrate metabolism, five in cofactor metabolism, and four in aromatic compound metabolism.

We also explored the frequency of fusions at the broadest level of the hierarchical classification supported by the SEED annotation ontology, subsystem class (Fig. 5b). Here, the classification is so broad that the level of variability is lower. However, we still found fusions occurring more often in some areas, specifically: (i) central metabolism, (ii) potassium metabolism, (iii) aromatic compounds, (iv) regulation and signaling, and (v) DNA metabolism.

### Distribution of predicted fusions among metabolic reactions

Next, we focused on patterns of fusions that occurred among genes annotated with metabolic functions. In this analysis, we will refer to a pair of fused genes coding for two enzymes, each one possessing a distinct functional role, as *fused roles*. We will also use the term *fused enzymes* to refer to the protein products of two fused genes which catalyze two distinct reactions. Our analysis of frequent fusions occurring in metabolism began with the 2,937 frequently fused functional roles identified in our large-scale fusion prediction algorithm. In this case, we used the mappings of reactions to functional roles in the ModelSEED resource [40]. We also used eight published microbial genome-scale metabolic models to associate specific biochemical reactions to metabolic functions that were in our frequently fused set. From this analysis, we were able to map 9,785 unique reactions to functional roles in the SEED annotations, of which 842 (7.1 %) were associated with functional roles that were frequently fused (Additional file 1: Table S16).

To understand why these specific reactions are more commonly associated with gene fusions, we used flux balance analysis on our eight published models to simulate growth in up to 520 growth conditions. We then classified reactions as essential (i.e. required for growth), active (i.e. present but not required for growth), or inactive (i.e. not present). We found that 1,703 (14 %) reactions were essential in at least one model for growth in at least one condition. Of these reactions, 172 were associated with frequently fused functional roles, which is 17 % of the total of reactions associated with frequently fused genes. Thus essential reactions are slightly over-represented among the reactions associated with frequently fused genes.

Similarly, our model analysis classified another 4,201 (34 %) reactions as active in at least one model during growth in at least one condition. Of these reactions, 335 are associated with frequently fused functional roles, which is 39.8 % of the total of reactions associated with frequently fused genes. Again, fusions are slightly over-represented in the set of active reactions.

We then sorted the reactions associated with fused enzymes by their associated standard Gibbs free energy change, as computed using the group contribution method [41]. This analysis revealed a number of reactions catalyzed by enzymes encoded by frequently fused genes that have highly positive free energy change values in the direction of flux (Additional file 1: Table S16).

**Fig. 6:**
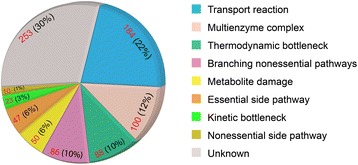
Functional analysis of frequently fused reactions. We identified 841 reactions as being frequently associated with gene fusion events. We manually assigned one of nine possible mechanistic explanations for the frequent fusion events associated with each of these reactions. The distribution of these mechanistic explanations is plotted as a pie chart (data extracted from Additional file 1: Table S16). Red numbers represent the number of reactions associated with a fusion event in a given category and the black numbers their respective percentages

Next we examined the average flux through all of our essential reactions across all our models and growth conditions. We found a number of reactions catalyzed by frequently fused roles that were associated with high flux values. Here we define high flux as flux in excess of 1 mmol/g CDW hr, or the same magnitude as the primary carbon source uptake in our FBA simulations, which is among the highest fluxes in metabolism. Complete results of the analysis of metabolic reactions associated with fusion events are summarized in Fig. 6 and shown in full in (Additional file 1: Table S16).

Finally, a total of 179 reactions associated with frequently fused roles were not active in any model in any growth condition. Hence, we had no data on flux or competing pathways for these fusions and were unable to formulate hypotheses concerning their formation.

### Fusions of neighboring genes and unstable metabolites

When analyzing our B vitamin enzyme fusions dataset, RibDd/RibDr and RibFa/RibFk emerged as a two pairs of neighbors in the riboflavin synthesis pathway with a high propensity to be fused (Fig. 2 and Additional file 1: Table S2). The intermediate 5-amino-6-(ribosylamino)-2,4-(1*H*,3*H*)-pyrimidinedione 5′-phosphate is a RibDd product and a RibDr substrate and is highly reactive [14]. Similarly, FMN is the RibFk product and the substrate for RibFa and, although less reactive than its precursors, if reduced to FMNH, becomes oxygen-sensitive [42]. On one hand it has been suggested that some fusions provide the infrastructure for tunneling or electrostatic channeling to prevent damage to reaction intermediates [43]. On the other hand, the channeling hypothesis has been recently challenged, since fusion *per se* did not promote channeling whereas formation of protein conglomerates did [29]. In view of these observations, we decided to search for fusions of genes that encode neighboring enzymes in metabolic pathways. A computational search for such fusions identified several genes that code for enzymes that produce or use unstable metabolites (Table 3). The results in this table are consistent with the fusion enrichments found in chorismate and heme synthesis pathways (Additional file 1: Table S14).

**Table 3:** Fusions of neighboring enzymes in metabolic pathways and their unstable substrates/products

Metabolism area	Enzyme roles	EC numbers	SEED gene identifier	Metabolite involved	References
Aromatic amino acids	Cyclohexadienyl dehydratase/Periplasmic chorismate mutase I precursor	4.2.1.51/5.4.99.5	fig|325240.9.peg.4134	Prephenate	[75, 76]
	Indole-3-glycerol phosphate synthase/Phosphoribosylanthranilate isomerase	4.1.1.48/5.3.1.24	fig|991999.3.peg.2431	1-(2-Carboxyphenylamino)-1-deoxyribulose 5-phosphate	[77]
Histidine	Phosphoribosyl-AMP cyclohydrolase/Phosphoribosyl-ATP pyrophosphatase	3.5.4.19/3.6.1.31	fig|751585.3.peg.1763	Phosphoribosyl-AMP	[78]
Glyoxalate	Isocitrate lyase / Malate synthase	4.1.3.1/2.3.3.9	fig|404589.10.peg.3099	Glyoxalate	[79–81]
Sulfur	Adenylylsulfate kinase/Sulfate adenylyltransferase subunit 1	2.7.1.25/2.7.7.4	fig|349163.14.peg.1814	Adenosine 5′-phosphosulfate	[82]
Folate	Aminodeoxychorismate lyase/Para-aminobenzoate synthase, aminase component	4.1.3.38/2.6.1.85	fig|257309.4.peg.1776	4-Amino-4-deoxychorismate	[83]
Phosphonate	2-Aminoethylphosphonate:pyruvate aminotransferase/Phosphonoacetaldehyde hydrolase	2.6.1.37/3.11.1.1	fig|691161.5.peg.2163	Phosphonoacetaldehyde	[84]
Siderophore	2,3-Dihydroxybenzoate-AMP ligase/Isochorismatase/Isochorismate synthase	2.7.7.58/3.3.2.1/5.4.4.2	fig|306537.3.peg.2089	Isochorismate	[85, 86]
Heme and siroheme biosynthesis	Precorrin-2 oxidase/Sirohydrochlorin ferrochelatase / Uroporphyrinogen-III methyltransferase	1.3.1.76/4.99.1.4/2.1.1.107	fig|644335.4.peg.2909	Precorrin 2	[87, 88]
	Uroporphyrinogen-III methyltransferase/Uroporphyrinogen-III synthase	2.1.1.107/4.2.1.75	fig|479834.4.peg.2988	UroporphyrinogenIII	[89–91]
	Porphobilinogen deaminase/Uroporphyrinogen-III synthase	2.5.1.61/4.2.1.75	fig|1049939.3.peg.1307	Hydroxymethylbilane	[92]

Fusions of genes encoding for neighboring enzymes were extracted from the SEED database computationally as described in Methods. The metabolites involved are products of one functional role cited in the row and substrates of the corresponding fused functional role. The References column gives citations documenting the chemical instability of the intermediates

### Integration of fusion data into an online web resource

All of the data from this large-scale fusion analysis have been loaded into an online web resource for browsing and searching: http://modelseed.org/projects/fusions/. This site includes seven tables: (i) a table of all genomes included in our analysis along with fusion counts in each genome; (ii) a table of all CDDs used in our analysis, along with CDD descriptions and predicted gene fusions associated with each CDD; (iii) a table of all CDD sets derived from our analysis, along with a list of all CDDs mapped into each set; (iv) a table of our complete *E. coli* and B vitamin fusion training sets, along with a source for each fusion and a list of the CDDs in each fusion; (v) a table of all functional roles with statistics on fusion frequency; (vi) a table of all SEED subsystems with statistics on fusion frequency; and (vii) a table of all predicted fusions along with a list of CDDs in each fusion. While these tables partially recapitulate Tables S9-S16, they add value in that they contain additional data that was impractical to include as supplementary material. The online version of the predicted fusion table (Additional file 3) is particularly useful given the large size of even a basic version of this table. All online tables can be sorted and queried by any field. These tables are particularly useful for mining our predicted fusions for insights relating to domains of unknown function as discussed in the supplementary material.

## Discussion and conclusions

In this work, we made multiple strides to enhance our understanding of protein fusions. First, we developed a highly curated training set of known fusions in *E. coli*, and more broadly in the B. vitamin pathways for a wide range of genomes. This work revealed the many difficulties involved in classifying genes in fusions, even in a well-studied organism like *E. coli*. No single previous approach or database provided a comprehensive list of fusions, and all previous datasets included numerous false positives. However, based on this analysis, we were able to use our curated training set to develop an improved fusion prediction algorithm that combines many of the strengths of previous approaches (see additional discussion in supplementary material). We then applied our new fusion prediction algorithm to predicting fusions for over 12 K genomes, permitting a global analysis of fusion events across all these genomes. This analysis showed that a large fraction of fusions involving metabolic enzymes. Many fusions involved two reactions with a shared substrate, pointing at either channeling [44] or coordination of complex formation [30] around a problematic intermediate metabolite. In other cases, we found fused enzymes at branch points in pathways, where fusion events could facilitate improved control of flux through such branch points. We also found many fusions comprised of subunits of multi-protein complexes. Our analysis also revealed enrichment for transport and regulatory proteins among gene fusion events, which could explain why potassium metabolism was specifically enriched in fusions, as it mainly contains transporter proteins. Finally, we found common fusion events in metabolism that revealed unexpected links between disparate metabolic pathways. Such fusions should be investigated as they might reflect cryptic relationships between metabolic functions. A deeper analysis of all of these findings, along with examples, are provided in the supplemental material.

**Fig. 7:**
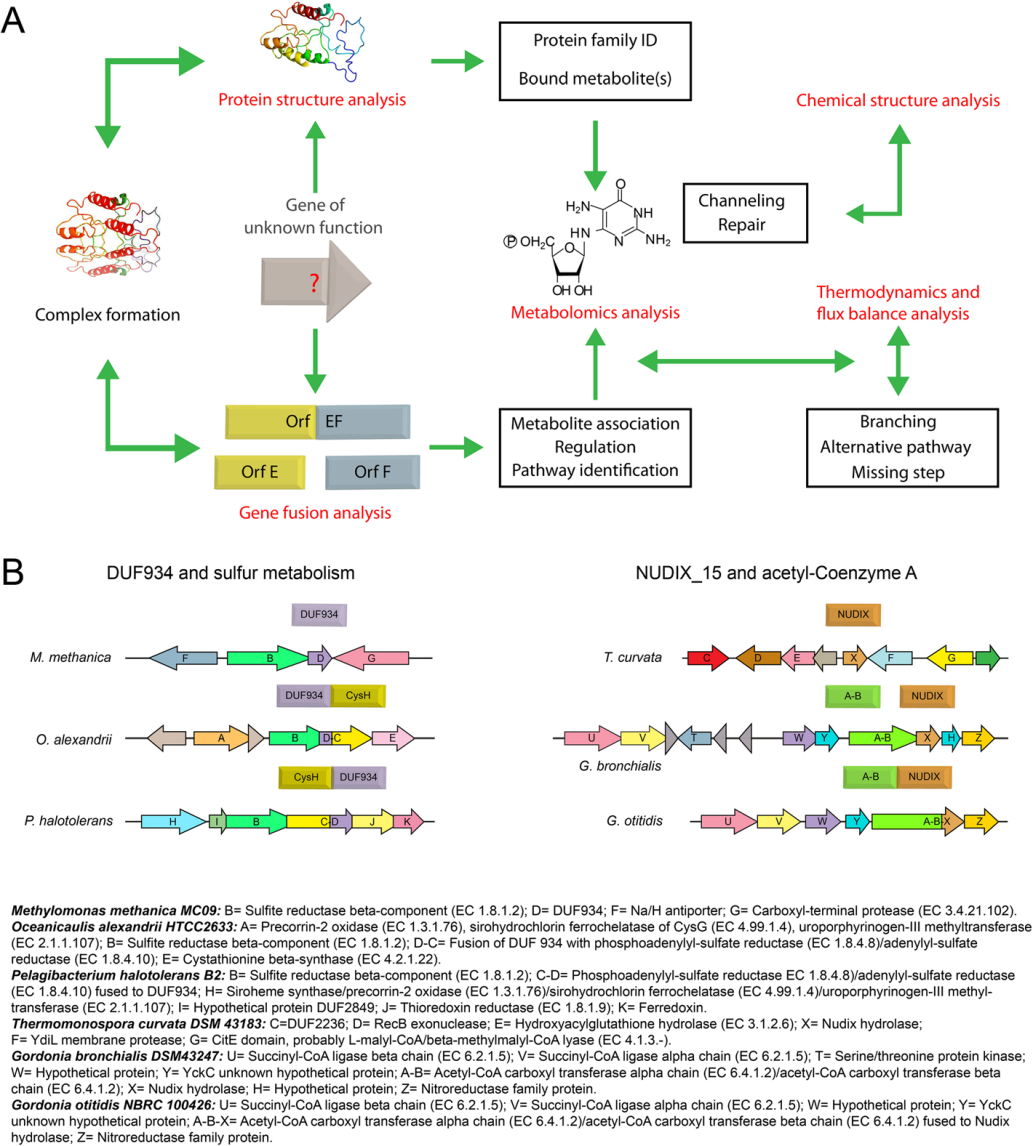
The use of fusions to infer functions of unknown domains. **a** Once a fusion of an unknown with a characterized gene is discovered, the function of the latter and the clustering pattern of the fusion gene help to propose functions for the unknown gene, especially when combined with structure analysis of the unknown and the fused product. If specific compounds are bound to the unknown protein and can be associated with the metabolic area of the known enzyme, mechanisms such as channeling or repair might be inferred. The position of the known enzyme in the pathway combined with flux balance and thermodynamics analysis can give clues about the function of the unknown gene. **b** Examples of the application of the ModelSEED fusions exploration tool. Beveled rectangles represent the genes that participate in the fusions used as starting points for our analysis. On the beveled rectangles, Cys H stands for phosphoadenylyl-sulfate reductase (EC 1.8.4.8)/adenylyl-sulfate reductase (EC 1.8.4.10); A-B stands for acetyl-coenzyme A carboxyl transferase alpha chain (EC 6.4.1.2)/acetyl-coenzyme A carboxyl transferase beta chain (EC 6.4.1.2); NUDIX stands for Nudix_15. These genes are also identified by the same color code as the arrows that represent them in the genome sections illustrated immediately below them. The rows of arrows depict the gene clustering areas given by the SEED platform for the genes analyzed in our examples. The genes in each organism’s genome section are represented by color coded arrows and identified by letters. The functional roles represented by these letters for each organism are given in the printed section below the illustration. Examples of the stand-alone genes and their clustering patterns are also given

Lastly, we found many cases where gene fusions events can provide insights into the function of previously un-annotated proteins. Many fusions have domains labeled only with COG (Clusters of Orthologous Groups) or DUF (Domain of Unknown Function) identifiers, yet something – perhaps much – about their function can be inferred from their strong association to a known functional role. As shown in Table 4, there are multiple cases where fusions between genes of unknown function and genes in a vitamin pathway led to the discovery of a novel function. We describe several examples in the supplementary material and in Fig. 7.

**Table 4:** Cases where a fusion of a domain of unknown function to a B vitamin gene led to a functional discovery

Domain	Vitamin pathway	Molecular function	ref
COG3236	Riboflavin	*N*-glycosidase	[14]
DUF89	CoA	Phosphatase	[93]
DUF1537	PLP	Kinase	[94]
Tnr3/Nudix	Thiamin	Pyrophosphatase	[13]
COG1058	Niacin	Pyrophosphatase	[95]
Human CoaD	CoA	Adenyl transferase	[10]
TenA-HAD	Thiamin	Hydrolase	unpublished
HAD-IA	Thiamin	Hydrolase	[96]
HAD-IB	Thiamin	Hydrolase	[96]

## Methods

### Manual collection and analysis of fusions

The *Escherichia coli* training set was developed by compiling fusions from four sources: Enright et al. [2], Serres et al. [33], IMG [34], and SEED [32] as described above. The Rosetta stone and conserved domain standards were applied using Conserved Domain Database (CDD) detection scripts given by NCBI [26]. We used three sources for the compilation of a representative set of B vitamin metabolism gene fusions: the NCBI protein conserved domain architecture retrieval tools [26], the HHMI Janelia Farm protein families architecture analysis tool [45], and SEED phylogenetic trees [27, 32]. Both the NCBI and HHMI architecture tools cover genomes in all kingdoms of life, but they rely only on sequence similarity. In this kind of analysis, all the paralogs of a gene that codes for a known enzyme are pooled together in a single type of fusion architecture, making it difficult to identify genes with fused domains of a specific function. On the other hand, in the SEED trees, fusions are flagged by a coloring system, making their detection possible within a phylogenetic as well as functional role context [32]. In our fusion search, for each functional role present in a particular B vitamin synthesis pathway, a representative gene was chosen in the model organism *E. coli* K12 MG1655. In the cases of genes which were absent in *E. coli*, the final choice of a suitable example was made after a search covering several organisms. After filtering fusion selections using the functional role and phylogeny criteria of SEED, they were analyzed with the protein family database Pfam [45] and the NCBI Conserved Domain Database [26] tools to confirm the presence of two domains with distinct functional roles.

In order to approach fusion analysis in a systematic fashion and to automate it, the custom software tool fusions.py was created. This tool catalogs all known fusion events occurring in a protein family of interest (or a set of families, e.g. in all enzymes of a vitamin biosynthesis pathway) by performing automatic batch search of the ‘Domain architecture’ collection of the Pfam database (http://pfam.xfam.org/search; [45]). Fusions.py uses as input a *.txt file with a list of query protein sequences in FASTA format (a single representative sequence per family is sufficient). For each input sequence the program identifies the corresponding Pfam protein family and queries its “Domain Architecture” data. The output file includes a list and a description of all fusion events (“architectures”) in which the corresponding family is involved. A single representative protein ID for each type of fusion events is listed. The code has been deposited at https://github.com/alekseyig/fusion.

### Counting B vitamin synthesis gene fusions, their variety and frequency

We separated the identified genes into two groups, the main role players and fusion partners. Main role players are genes belonging to each specific B vitamin synthesis canonical pathway that occur in the widest variety of fusions. We used these as focus points for analysis. We classified fusion partners in three categories: genes from each specific B vitamin pathway (including those for repair and recycling enzymes, regulators and repressors), genes from other areas of metabolism, and unknown genes (Additional file 1: Tables S2A-S8A). We counted the number of fusion events of each specific B vitamin pathway gene with other genes in each of the three categories above). This is the number of instances that each specific gene appears in all the three domain columns of the respective B vitamin gene table see Additional file 1: Tables S2A-S8A). We took this number of architectures as a measure of the variety of fusion events in which each B vitamin gene participates and entered this number in the “Number of binary fusion events” column of the corresponding B vitamin genes table (see Additional file 1: Tables S2B-S8B).

A representative set of ~1,000 diverse prokaryotic genomes in the SEED database (created as described below or in [46, 47]) was scanned to account for all cases when each of the B vitamin synthesis genes was present in this group sample and also the instances when this specific gene participated in a fusion event of any type (Additional file 1: Tables S2B-S3B). The frequency was then expressed as a percentage and calculated as the ratio of the number of fusions in which each vitamin synthesis gene participated within the pool of ~1,000 genomes divided by the number of representatives of this specific gene present in this pool (see column of “total proteins annotated with this role” in Additional file 1: Tables S2B and S3B). We considered the resultant ratios as representatives of the frequency with which each specific B vitamin synthesis gene is found fused in prokaryotes. Note, however, that this is a relative ratio because a given gene might be present in more than a single copy in an individual genome and might be entirely absent in some bacterial taxa.

### Representative set of ~1000 diverse prokaryotic genomes in the SEED database

With approximately 30,000 prokaryotic genomes currently available in public databases and many more in the pipeline (www. genomesonline.org), it was not practical to perform meaningful comparative analysis on all of them simultaneously. Thus, the algorithm for computing molecular operational taxonomic units (OTUs) based on DNA barcode data [48, 49] was used to group the 12,600 prokaryotic genomes available in the SEED database into about 1,000 taxon groups. A representative genome for each OTU was selected based on the largest amount of published experimental data and the highest level of research interest within the scientific community for different microorganisms within each OTU. The resultant collection of 983 diverse eubacterial and archaeal genomes creates a manageable set that accurately represents the immense diversity of the prokaryotes with sequenced genomes in the SEED database. Importantly, it is not skewed by an overabundance of genomes for a handful of medically or industrially important microbial genera such as enterobacteriaceae, staphylococci, and mycobacteria.

### Use of metabolic models to evaluate reaction activity and essentiality

Flux balance analysis [50, 51] was used in combination with eight published genome-scale metabolic models [38, 52–58] to produce a database of metabolic reactions, along with associated predicted essentiality and activity. Models were selected to represent eight diverse organisms, including one yeast [59] and seven bacteria [38, 52–58]. Growth was simulated on over 520 growth conditions (including various minimal media [60] and rich media such as LB and BHI), with flux variability analysis [61] applied in each condition to identify active and essential reactions in all models. Reactions were classified as active in a particular growth condition if they could carry flux but did not have to carry flux in order for biomass production to occur. Reactions were classified as essential in a particular growth condition if they had to carry flux in order for biomass production to occur.

### Thermodynamics

The thermodynamics analysis of the reactions was made calculating the associated standard Gibbs free energy change, as computed using the group contribution method [41].

## Abbreviations

OTU, operational taxonomic unit; CDD, conserved domain database

## Additional files

Additional file 1: Is an excel file containing Supplemental **Tables S1**-**S16**. (XLSX 11230 kb) Additional file 2: Is a discussion of how fusions are distributed among the B. vitamin pathways, based on our manual curation and a discussion on the most prevalent fusions. (DOCX 57 kb) Additional file 3: Is a zip archive containing a tab-delimited table of all 3.8 million predicted fusions. (TXT 494892 kb)
