# Supplementary material for: Systematic identification and analysis of frequent gene fusion events in metabolic pathways
Source: BMC Genomics. 2016 Jun 24;17:473. doi: 10.1186/s12864-016-2782-3 (PMC4921024; doi:10.1186/s12864-016-2782-3)
Supplement: Additional file 2: — Is a discussion of how fusions are distributed among the B. vitamin pathways, based on our manual curation and a discussion on the most prevalent fusions. (DOCX 57 kb) [file 12864_2016_2782_MOESM2_ESM.docx]

**Detailed discussion of B vitamin pathway fusions.**

Fusions of B vitamin pathways genes showed distinct patterns in their distribution, frequency, and variety. Fusions followed different distribution patterns along the biosynthetic pathways (Figures 2 and 3). Most of the genes corresponding to each pathway were involved in at least one fusion event, except for the pyridoxal phosphate genes (Tables S2-S8 in Additional file 1). Some proteins like RibB, RibA, ThiE, ThiD, (Figures 2 and 3 and Tables S2B-S3B in Additional file 1), CoaE, FolB, and FolK (Tables S4B-S8B in Additional file 1) participated in more than seven different fusion events. Some points in these pathways were hot spots for fusions (Figures 2 and 3); possible rationales for this will be discussed later.

Some of the B vitamin gene fusions analyzed were extremely frequent (Figures 2 and 3 and Tables S2 and S3 in Additional file 1). For example, RibFa/RibFk and RibDd/RibDr were fused in around 99% of the genomes analyzed. Another pair, RibA/RibB, were fused in 77% of cases. The most prevalent fusions involved functions catalyzing adjacent steps in a pathway, such as RibDd/RibDr or RibFa/RibFk. However, not all genes encoding adjacent steps in the riboflavin pathway were found in fusions. For instance, RibH and RibE were fused in only ~0.5% of all genomes analyzed (Figure 2 and Table S2B in Additional file 1).

B vitamin genes participated in fusions with genes in the same or different pathways. The most common fusions were between functions in the same pathway. In the case of the riboflavin pathway, out of the 22 fusion types, 13 involved genes within the same pathway, four involved well annotated genes of other pathways, and nine involved poorly characterized domains (Figure 2 and Table S2A in Additional file 1). Similarly, for thiamin, 20 out of 25 fusion types involved genes within the same pathway (Fig. 3 and Table S3A in Additional file 1). A substantial number of B vitamin synthesis proteins were fused to domains of unknown function (Tables S2-S8 in Additional file 1). This is particularly important, since our fusion detection algorithm aims to detect fusions of well-characterized genes with domains of unknown function.

**Refining the definition of a gene fusion to build a new fusion prediction algorithm**

Our comparison of the previously reported *E. coli* fusions datasets revealed the difficulties in classifying genes as fusions, even in a well-studied organism. No single method gave a comprehensive list and manual analysis and expert judgment had to be applied to all datasets. The combination of curated sets of known fusions from the B vitamin pathways and the *E. coli* genome permitted the development of a refined set of specific criteria to define a fusion event, which merged elements of the existing domain-based and Rosetta-stone-protein-based fusion definitions. These criteria formed the basis for a new high-speed fusion prediction algorithm, which we applied to generate a vast dataset of 3.8 million potential fusions across ~12,000 genomes. This new fusion dataset gives insights into why fusions occur, and is a valuable resource to support the functional annotation of genes included in the dataset.

**Analysis of fusion events in metabolism**

Our global analysis of fusion events showed that a large fraction of fusions involve metabolic enzymes. A study of the reactions associated with these enzymes suggested that many fusions occur in order to overcome thermodynamic and kinetic bottlenecks in metabolic pathways, or simply to optimize the flux through central pathways that have a large impact on growth rate, and thus cell fitness. Channeling is one major mechanism for overcoming these bottlenecks in metabolic pathways, but of course not all fused proteins have a structure that can promote channeling. Hence, a careful structural analysis of the proteins involved in a fusion should be done to test the channeling hypothesis. Moreover, channeling can also be supported by separate proteins arranged into an enzymatic complex as in bacterial tryptophan synthase [[1](#_ENREF_1)]. Indeed, fusions can be a contributing element to complex formation, as cited above [[2](#_ENREF_2)]. In conclusion, detecting fusions of neighboring functions can be used in the search for channeling mechanisms but is only a starting point.

In other cases, we found that fused enzymes are commonly located at branch points in pathways, where fusion events could either prevent the consumption of an intermediate metabolite by a competing side pathway, or facilitate the coordinated production of two metabolites that then react with each other in a subsequent pathway step. A prominent example is the fusion of RibA (rxn05040, Table S16 in Additional file 1) to RibB (rxn00300, Table S16 in Additional file 1) in riboflavin synthesis. The RibA- and RibB-mediated reactions are not consecutive steps in a pathway, but rather produce two intermediates that are subsequently combined in a later step. A more complicated case is the fusion of three enzymes of thiamin biosynthesis (Figure 3 and Table S3 in Additional file 1): (i) TenI, a thiazole tautomerase, that produces 2-(2-carboxy-4-methylthiazol-5-yl)ethyl phosphate; (ii) ThiD, a hydroxymethylpyrimidine phosphate kinase that produces 4-amino-2-methyl-5-diphosphomethylpyrimidine; and (iii) ThiE, a thiamin-phosphate pyrophosphorylase, that consumes the products of TenI and ThiD to produce thiamin monophosphate. In both examples, a fusion could help co-localize intermediates to facilitate and coordinate the coupling of cellular processes that must occur in parallel.

Another common source of fusions is the combination of enzymes comprising a multi-protein complex. One example of this type is the CoA synthesis enzyme phosphopantothenoylcysteine synthetase, CoaBC (rxn02341, Table S16 in Additional file 1), whose domains, in other organisms, exist as separate proteins that form a complex [[3-5](#_ENREF_3)]. This enrichment of fused proteins that form complexes has been noted in other studies [[2](#_ENREF_2)].

Still another reason for metabolic fusion events could be to reduce the potential detrimental effects of metabolite damage. The fusion of neighboring enzymes in pathways can sometimes be correlated with instability of intermediates (Table 4); known cases in which fusions support the channeling of unstable metabolites include formiminotransferase/cyclodeaminase [43] , yeast tryptophan synthase [[1](#_ENREF_1)], and proline dehydrogenase /1-pyrroline-5-carboxylate dehydrogenase [[6](#_ENREF_6)]. Fusions may also link enzymes that produce unstable compounds to enzymes that destroy an excess of the unstable compound or its breakdown products [[7](#_ENREF_7)]. In other cases, the fused enzymes work together to form a repair system to restore a damaged metabolite to its pristine condition, as is the case for the bacterial NAD(P)H hydrate epimerase/NAD(P)H hydrate dehydratase [[8](#_ENREF_8)]. Such fusions form an important element of the network of housecleaning and repair enzymes in the cell [[9](#_ENREF_9)], which is why fusion analysis is useful for discovering metabolite damage-repair systems (Table 4).

The fusion events in metabolism also revealed unexpected links between disparate metabolic pathways. We found three examples of this type of fusion among the B vitamin pathways: (i) ACAD/RibA, a fusion of acyl-Coenzyme A dehydrogenase and GTP cyclohydrolase II (Table S2 in Additional file 1); (ii) TruB/RibFa/RibFk, a fusion of tRNA pseudouridine synthase B/FMN adenylyltransferase/riboflavin kinase (Table S2 in Additional file 1); (iii) RibFa/RibFk/MnmA, a fusion of FMN adenylyltransferase/riboflavin kinase/tRNA-specific 2-thiouridylase (Table S2 in Additional file 1); and (iii) BirA/CoaX a fusion of biotin-protein ligase with pantothenate kinase type III (Table S4 in Additional file 1). Inter-pathway fusions also occur in eukaryotes (e.g. the fusion of pyridoxamine 5'-phosphate oxidase and NAD(P)H-hydrate epimerase in plants [[10](#_ENREF_10)]). Such fusions should be investigated as they might reflect cryptic relationships between metabolic functions.

**Analysis of fusion events in transporters and regulators**

Our global fusion analysis also revealed enrichment for transport and regulatory proteins among gene fusion events. This was true both in the *E. coli* set (Figure 1, Table S1 in Additional file 1) and in the total fusion set (Figure 5, Table S14 in Additional file 1). In the case of the manually curated B vitamin set, these fusions were not captured by design as we focused only on the genes that were fused to synthesis enzymes. However, fusions in transporters can also be found in the B vitamin area. For example, the thiamin ABC transporter (rxn09297, Table S16 in Additional file 1) is composed of transmembrane subunits, substrate-binding subunits, and ATP hydrolysis subunits that are often fused. This fusion enrichment in transport proteins could also explain why the potassium metabolism area was specifically enriched in fusions, as it mainly contains transporter proteins.

**Fusions of well characterized genes with unknowns**

The fusions identified in this work are a valuable resource for future genome annotation, particularly for the large number of fusions involving domains for which no annotation exists. For example, many fusions have domains labeled only with COG (Clusters of Orthologous Groups) or DUF (Domain of Unknown Function) identifiers, yet something – perhaps much – about their function can be inferred from their strong association to a known functional role. As shown in Table 4, there are multiple cases where fusions between genes of unknown function and genes in a vitamin pathway led to the discovery of a novel function. Of particular interest are the domains from the Nudix [[11](#_ENREF_11)] or HAD (HaloAcid Dehalogenase [[12-14](#_ENREF_12)]) superfamilies, as their general function (pyrophosphatase or phosphatase, respectively) can be inferred with reasonable confidence. The fusion dataset produced here is a valuable and rich resource to mine these associations. Indeed, searching for the term ‘DUF’ in the fusion list gave ~300,000 fused genes (of course not all independent). When searching for more specific domains, ~8,000 genes were found to have a fusion with a Nudix hydrolase domain. Two examples of such fusions, one with a DUF and one with a Nudix domain, and how these can be used to predict function, are discussed below.

The first example links DUF934 and sulfur metabolism (Figure 7B). Cells use a large amount of energy in assimilating sulfate into cysteine, homocysteine, and other sulfur compounds. This energy demand creates selective pressure to salvage or repair metabolites containing activated or reduced sulfur [[15](#_ENREF_15)]. To hunt for potential salvage or repair enzymes in sulfur metabolism, we analyzed fusions involving sulfur metabolism genes and found eight cases of fusions of phosphoadenylyl-sulfate reductase (CysH) (EC 1.8.4.8), one of the first enzymes in sulfate assimilation, with DUF934. These fusions occurred in both possible orientations: CysH/DUF934 and DUF934/CysH. Moreover, analysis of the clustering pattern of these fusions in SEED showed that both types of fusion, as well as stand-alone DUF934 encoding genes, cluster with sulfite reductase genes (Figure 7B). Since both phosphoadenylyl-sulfate and adenylyl-sulfate are quite reactive and can undergo chemical damage [[16](#_ENREF_16)], it is reasonable to propose that DUF934 participates in the salvage or repair of activated sulfate compounds.

The second fusion example is between the central metabolism enzyme acetyl-coenzyme A carboxyl transferase (aCoACT) (EC 6.4.1.2), and a protein belonging to subgroup 15 (or cd04673 CDD domain) of the Nudix superfamily [[11](#_ENREF_11)]. aCoACT is one of the first enzymes of fatty acid synthesis [[17](#_ENREF_17)]. This protein occurs in >2,000 gene fusion events of which the majority are fusions between the alpha and beta subunits of this enzyme as well as fusions with biotin carboxylase that forms part of the same enzyme complex [[17](#_ENREF_17)]. By limiting the search to fused genes ranging from 1,500 to 2,000 base pairs in size, we identified a Nudix domain fused to aCoACT in various species of *Gordonia*. As Nudix enzymes are known to hydrolyze coenzyme A [[18](#_ENREF_18)] and acyl-coenzyme A [[19](#_ENREF_19)], it is possible that Nudix subfamily 15 hydrolyzes acetyl-coA or some modified form of this compound. We then used SEED to explore the clustering patterns of the fusion in *Gordonia polyisoprenivorans* HW436 and its closest homologs. Fused genes of aCoACT/Nudix_15 were found to cluster with genes encoding the alpha and beta chains of the TCA cycle enzyme succinyl-CoA ligase (Figure 7B). Members of the Nudix_15 subfamily were also found to occur as stand-alone proteins in several organisms, and in some cases to cluster with succinyl-CoA ligase alpha and beta chains or with L-malyl-CoA/beta-methylmalyl-CoA lyase (Figure 7B). Thus fusion analysis points to a role for the Nudix_15 subfamily in the hydrolysis of acetyl-coA or a derivative thereof.

**References**

1. Huang X, Holden HM, Raushel FM: **Channeling of substrates and intermediates in enzyme-catalyzed reactions**. *Annu Rev Biochem* 2001, **70**:149-180.

2. Marsh JA, Hernandez H, Hall Z, Ahnert SE, Perica T, Robinson CV, Teichmann SA: **Protein complexes are under evolutionary selection to assemble via ordered pathways**. *Cell* 2013, **153**(2):461-470.

3. Daugherty M, Polanuyer B, Farrell M, Scholle M, Lykidis A, de Crécy-Lagard V, Osterman A: **Complete reconstitution of the human coenzyme A biosynthetic pathway via comparative genomics**. *J Biol Chem* 2002, **277**(24):21431-21439.

4. Strauss E, Kinsland C, Ge Y, McLafferty FW, Begley TP: **Phosphopantothenoylcysteine synthetase from *Escherichia coli*. Identification and characterization of the last unidentified coenzyme A biosynthetic enzyme in bacteria**. *J Biol Chem* 2001, **276**(17):13513-13516.

5. Bucovaz ET, Macleod RM, Morrison JC, Whybrew WD: **The coenzyme A-synthesizing protein complex and its proposed role in CoA biosynthesis in bakers' yeast**. *Biochimie* 1997, **79**(12):787-798.

6. Singh H, Arentson BW, Becker DF, Tanner JJ: **Structures of the PutA peripheral membrane flavoenzyme reveal a dynamic substrate-channeling tunnel and the quinone-binding site**. *Proc Natl Acad Sci U S A* 2014, **111**(9):3389-3394.

7. Frelin O, Huang L, Hasnain G, Jeffryes JG, Ziemak MJ, Rocca JR, Wang B, Rice J, Roje S, Yurgel SN *et al*: **A directed-overflow and damage-control N-glycosidase in riboflavin biosynthesis**. *Biochem J* 2014.

8. Marbaix AY, Noel G, Detroux AM, Vertommen D, Van Schaftingen E, Linster CL: **Extremely conserved ATP- or ADP-dependent enzymatic system for nicotinamide nucleotide repair**. *J Biol Chem* 2011, **286**(48):41246-41252.

9. Galperin MY, Moroz OV, Wilson KS, Murzin AG: **House cleaning, a part of good housekeeping**. *Mol Microbiol* 2006, **59**(1):5-19.

10. Niehaus TD, Richardson LG, Gidda SK, ElBadawi-Sidhu M, Meissen JK, Mullen RT, Fiehn O, Hanson AD: **Plants utilize a highly conserved system for repair of NADH and NADPH hydrates**. *Plant Physiol* 2014, **165**(1):52-61.

11. McLennan AG: **The Nudix hydrolase superfamily**. *Cell Mol Life Sci* 2006, **63**(2):123-143.

12. Koonin EV, Tatusov RL: **Computer analysis of bacterial haloacid dehalogenases defines a large superfamily of hydrolases with diverse specificity. Application of an iterative approach to database search**. *J Mol Biol* 1994, **244**(1):125-132.

13. Burroughs AM, Allen KN, Dunaway-Mariano D, Aravind L: **Evolutionary genomics of the HAD superfamily: understanding the structural adaptations and catalytic diversity in a superfamily of phosphoesterases and allied enzymes**. *J Mol Biol* 2006, **361**(5):1003-1034.

14. Kuznetsova E, Proudfoot M, Gonzalez CF, Brown G, Omelchenko MV, Borozan I, Carmel L, Wolf YI, Mori H, Savchenko AV *et al*: **Genome-wide analysis of substrate specificities of the *Escherichia coli* haloacid dehalogenase-like phosphatase family**. *J Biol Chem* 2006, **281**(47):36149-36161.

15. Sekowska A, Danchin A: **Sulfur metabolism in Bacteria, with emphasis on *Escherichia coli* and *Bacillus subtilis***. In*.*; 2000: <http://www.normalesup.org/~adanchin/science/sulfur-review.html>.

16. Schmidt A, Jager K: **Open questions about sulfur metabolism in plants.** *Annu Rev Plant Physiol Plant Mol Biol* 1992, **43**:325-349.

17. Barber MC, Price NT, Travers MT: **Structure and regulation of acetyl-CoA carboxylase genes of metazoa**. *Biochim Biophys Acta* 2005, **1733**(1):1-28.

18. AbdelRaheim SR, McLennan AG: **The *Caenorhabditis elegans* Y87G2A.14 Nudix hydrolase is a peroxisomal coenzyme A diphosphatase**. *BMC Biochem* 2002, **3**:5.

19. Reilly SJ, Tillander V, Ofman R, Alexson SE, Hunt MC: **The nudix hydrolase 7 is an Acyl-CoA diphosphatase involved in regulating peroxisomal coenzyme A homeostasis**. *J Biochem* 2008, **144**(5):655-663.
